# Attention-Guided Image Captioning through Word Information

**DOI:** 10.3390/s21237982

**Published:** 2021-11-30

**Authors:** Ziwei Tang, Yaohua Yi, Hao Sheng

**Affiliations:** School of Printing and Packaging, Wuhan University, Wuhan 430072, China; tangziwei@whu.edu.cn (Z.T.); 2015301750041@whu.edu.cn (H.S.)

**Keywords:** image captioning, word level attention, previous word guidance, current word guidance

## Abstract

Image captioning generates written descriptions of an image. In recent image captioning research, attention regions seldom cover all objects, and generated captions may lack the details of objects and may remain far from reality. In this paper, we propose a word guided attention (WGA) method for image captioning. First, WGA extracts word information using the embedded word and memory cell by applying transformation and multiplication. Then, WGA applies word information to the attention results and obtains the attended feature vectors via elementwise multiplication. Finally, we apply WGA with the words from different time steps to obtain previous word guided attention (PW) and current word attention (CW) in the decoder. Experiments on the MSCOCO dataset show that our proposed WGA can achieve competitive performance against state-of-the-art methods, with PW results of a 39.1 Bilingual Evaluation Understudy score (BLEU-4) and a 127.6 Consensus-Based Image Description Evaluation score (CIDEr-D); and CW results of a 39.1 BLEU-4 score and a 127.2 CIDER-D score on a Karpathy test split.

## 1. Introduction

Image captioning is synthetic research that spans computer vision and natural language processing to generate natural descriptions of images. In recent years, image captioning has made great progress with the rapid development of classification [1], object detection [2], and machine translation. However, there are many problems, such as object recognition and interactions, and corresponding relations between objects and words, making it a challenging task [3,4,5,6,7].

Inspired by attention mechanisms [8] and sequence–sequence models [9] exploited in machine translation tasks, an encoder–decoder framework [10,11,12,13,14] has been widely used for image captioning. In such a framework, images are encoded to feature vectors by a pretrained image classification model, object detection model, or semantic segmentation model, and then decoded to words via an RNN. Within the RNN network [15], the decoder process is implemented as a sequence to generate words one by one. Until the attention mechanism was proposed, there was little optimization for this framework. The attention mechanism [16], which comes from machine translation, can guide the generation of words by weighting a feature to connect a portion of an image with a word at each time step.

**Figure 1:**
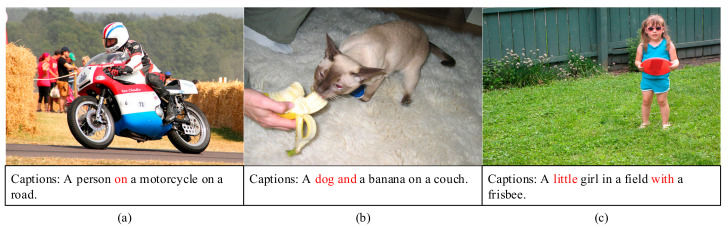
Illustration of some defects that attention may produce without guidance. We present some of them, such as a simplistic logical description (**a**), incorrect object recognition (**b**), and poor details of a target (**c**). The words in red are the incorrect issues.

Currently, an attention mechanism is widely applied in image captioning [17] systems. Because the attention directly determines the caption of an image, the inference direction determined by attention modules must be correct. However, attention is concentrative and superficial in general. It is prone to results in the decoder knowing little or mistaking the objects of the image, such as the “*dog*” in Figure 1b. In detail, the decoder may be misled to list nouns into generated sentences simply and ignore the relationships among the objects, for example the relation between “*person*” and “*motorcycle*” in Figure 1a and what the “*dog*” is doing in Figure 1b. Moreover, an attended region only represents one word, which means that the decoder may overlook the details of an object, for instance the word “*little*” depicting the “*girl*” in Figure 1c.

To address this issue, we propose word guided attention (WGA), which is created from word information, to bring novel specific guidance to the decoder. First, we design new information processing for words with several transformations and activation functions, which is similar to GLU [18]. The information processing method includes memory cell weighting, embedded words, and basic attention. Based on this process, we construct a WGA module in the decoder. Subsequently, we utilize the WGA and propose respective methods for the different step words. Fused with the previous step word, the previous word guided attention (PW) can be achieved. In addition, the current step word constitutes the current word guided attention (CW).

In this paper, we apply self-attention [19] as the basic attention unit for both the encoder and decoder phases. In the encoder, self-attention is used to build relations among the objects by weighting feature vectors extracted from an image. In the decoder, self-attention points out the major objects in an image and plays a guided role in PW/CW. Furthermore, we propose PW to expand the scope of objects describing and intensify the relationships via word-level attention. On the other hand, CW is concentrated on the current saliency region to obtain more detailed content and deeper relations.

We evaluate our method on the MSCOCO dataset and perform quantitative and qualitative analysis. The results show that our WGA is effective. The proposed PW/CW model is superior to other published image caption models. The main contributions of our paper include the following:

- We propose a novel word guided attention module for image captioning to determine the relationships among the attention features of an encoded image.
- We use the WGA with the previous step word and the current step word. With the previous word, the WGA concentrates on covering more objects in the scene and describing the relevance among them. With the current step word, the WGA is devoted to obtaining more details and deeper relation information from the current attention region.

## 2. Related Work

### 2.1. Image Captioning

Recent image captioning approaches are based on the framework of an encoder–decoder, which benefits from the development of deep learning and machine translation [8]. For example, an end-to-end CNN-LSTM framework is proposed to encode an image into CNN feature vectors and decode them into a sentence [20]. In [21], high-level semantic information is proposed as a CNN-LSTM framework. In [22], a two-layer LSTM is applied to give attention to a performing stage. Moreover, some complicated information, such as attributes and relationships, is integrated to improve the generated captions covering an image more completely [23,24,25].

### 2.2. Attention Mechanism

The attention mechanism [26], which originates from simulating the human perception system, has been widely employed and has made a great progress for seq-to-seq tasks. In image captioning, attention is an essential part of the model. In [16], a weighted candidate vector is proposed to teach the decoder focusing on the right fields in an image using normalization and SoftMax function. Since then, many studies on attention mechanisms have emerged for image captioning, such as adaptive attention [27], Sca-cnn [28].

In machine translation, we can obtain much inspiration for image captioning. For instance, novel attention achieved from the words is proposed in [29]. In [19], self-attention is proposed and obtains state-of-the-art results.

## 3. Methods

We first introduce the WGA module. Then we present how the WGA works for different image captioning phases.

### 3.1. WGA

**Figure 2:**
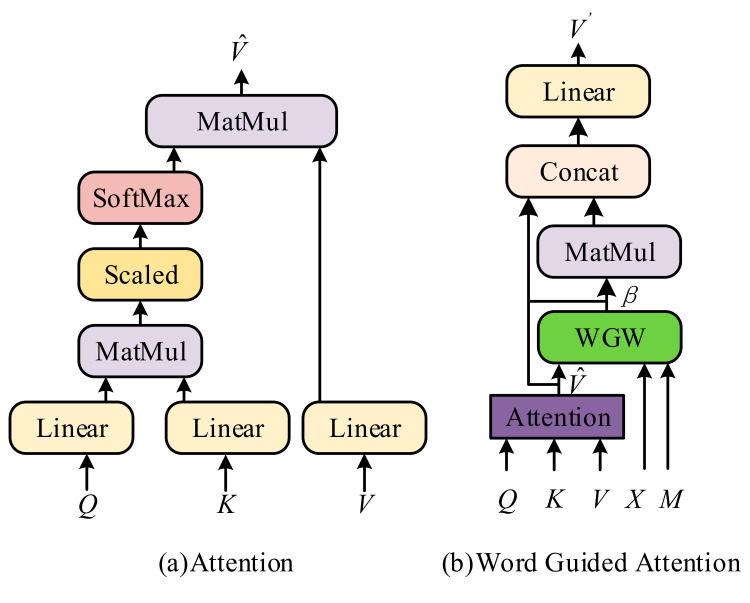
Illustration of a basic attention unit and WGA. (**a**) This unit produces the average weighted feature vectors by linear transformation, scaled dot-product attention, and SoftMax. (**b**) The WGA combines attention with word guided weighting (WGW) via elementwise multiplication.

A basic attention unit $f_{\mathrm{Att}}\left(.\right)$ provides the weighted feature vectors $\hat{V}$ for queries, keys, and values (denoted as *Q*, *K,* and *V*, respectively) by some operations, as shown in Figure 2a. First, we operated on *Q*, *K*, and *V* to linearize them independently. Then, the similarity weight between *Q* and *K* was measured by the dot-product, exerting scale correction and the SoftMax function. Finally, we performed matrix multiplication between the similarity weight and *V*. Thus, a basic attention unit $\hat{V}=f_{\mathrm{Att}}\left(Q,\;K,\;V\right)$ can be formulated as

(1) $$sim_{i,j}=\left(W_{Q}q_{i}+b^{Q}\right)⊙\left(W_{K}k_{j}+b^{K}\right),$$

(2) $$\alpha_{i,j}=\frac{sim_{i,j}}{\sqrt{d_{k_{j}}}},$$

(3) $${\hat{v}}_{i}=\sum_{j}\frac{e^{\alpha_{i,j}}}{\sum_{j}e^{\alpha_{i,j}}}\left(W_{V}v_{j}+b^{V}\right),$$

where $q_{i}\in Q$, $k_{j}\in K$, and $v_{j}\in V$, $W_{Q}{,\;W}_{K}{,\;W}_{V}\in {\mathbb{R}}^{D\times D},\;b^{*}\in {\mathbb{R}}^{D}$ are the linear transformation, bias of queries, keys, and values respectively, and *D* is the dimension of a vector. *sim_i_*_,*j*_ denotes a function to calculate the similarity score between $q_{i}\;$ and $k_{j}\;$ via the dot-product ⊙, and ${\hat{v}}_{i}\in \hat{V}$ is the attended feature vector.

A basic attention unit outputs the preliminarily attended feature vectors, which can direct a language model to generate more nouns and be effective building their relationships. However, it is too compulsive to obtain correlations among irrelevant objects and may ignore some inconspicuous objects.

Therefore, we proposed the WGA module $f_{\mathrm{WGA}}\left(.\right)$, as shown in Figure 2b, to extract guiding information from a generated word. The WGA module generates a word guiding weighting (WGW) $\beta_{i}$, which is extremely conditioned on the attention unit result $\hat{V}$ and adopts elementwise multiplication for $\beta_{i}$ and $\hat{V}$ to output word guided attended feature vectors $V^{\prime }$ using a residual connection. $V^{\prime }=f_{\mathrm{WGA}}\left(Q,\;K,\;V,\;X,\;M\right)$ can be determined by

(4) $$\beta_{i}=f_{WGW}\left({\hat{v}}_{i},W_{e}x,\;m_{T}\right),$$

(5) $${v^{\prime }}_{i}=W_{\lambda }\left\{\left[{\hat{v}}_{i},\;\left(W_{\phi }\left({\hat{v}}_{i}\cdot \beta_{i}\right)+b^{\phi }\right)\right]+b^{\lambda }\right\},$$

where $v_{i}^{\prime }\in V^{\prime }$, $f_{\mathrm{WGW}}\left(.\right)$ denotes the process of *WGW*, [., .] is a function for concatenating two vectors, x ∈ X defines the embedded word, $m_{T}\in M$ is a memory cell, and T is the set of time steps. $W_{\lambda }{,\;W}_{\phi }\in {\mathbb{R}}^{D\times D},\;b^{*}\in {\mathbb{R}}^{D}$ are two linear transformation matrixes and $W_{e}$ is the word embedding matrix.

**Figure 3:**
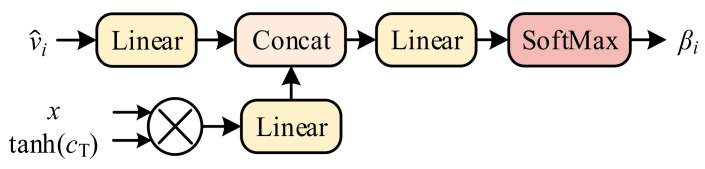
The WGW module in the WGA, where the embedded word and memory cell refine the feature vectors by strengthening the connection with existing words.

Then, the WGW $f_{\mathrm{WGW}}\left(.\right)$ is central to the WGA to obtain the guiding weight information from a word. As shown in Figure 3, WGW first employs a memory cell *M* and the individual word *X* to strengthen the influence of generated words for the subsequent sentence content via an activation function and elementwise multiplication. Then, WGW utilizes linear transformation and merges word context information $g_{T}^{x}$ with ${\hat{v}}_{i}$ Finally, the SoftMax function is used to obtain the weighting $\beta_{i}.$ $\beta_{i}=f_{\mathrm{WGW}}\left({\hat{v}}_{i},\;W_{e}x_{T},\;m_{T}\right)$ and can be represented as

(6) $$g_{T}^{x}=W_{e}x_{T}\tanh\left(m_{T}\right),$$

(7) $${\hat{\beta }}_{i}=W_{{\hat{\beta }}_{i}}\tanh\left(\left[W_{\hat{v}}{\hat{v}}_{i}+b^{\hat{v}},\;W_{g}g_{T}^{x}+b^{g}\right]\right)+b^{{\hat{\beta }}_{i}},$$

(8) $$\beta_{i}=\frac{e^{{\hat{\beta }}_{i}}}{\sum_{i}e^{{\hat{\beta }}_{i}}},$$

where $W_{{\hat{\beta }}_{i}}{,\;W}_{\hat{v}},\;W_{g}\in {\mathbb{R}}^{D\times D},\;\mathrm{and}\;b^{*}\in {\mathbb{R}}^{D}$ are matrixes of linear transformation and corresponding biases. The tanh denotes an activation function.

### 3.2. Image Captioning Model

We took advantage of the WGA for image captioning model based on the two-LSTM encoder–decoder framework.

For an image *I*, CNN feature vectors $A=\left\{a_{1},a_{2},\ldots ,a_{n}\right\}$ are extracted, where $a_{n}\in {\mathbb{R}}^{D},\;n\in N$ and *N* is the number of feature vectors in the image. In encoder phase *Enc*, we not only obtained the feature vectors, but also fed *A* to the basic attention unit (Figure 2a) $f_{\mathrm{Att}}\left(Q,\;K,\;V\right)$, where *Q*, *K* and *V* are inputs of *A*. *Enc* can be formulated as

(9) $${\hat{A}}_{E}=Enc\left(f_{Att},\;A^{Q},\;A^{K},\;A^{V}\right),$$

where ${\hat{A}}_{E}=\left\{{\hat{a}}_{1},{\hat{a}}_{2},\ldots ,{\hat{a}}_{n}\right\}$ is the encoded result of an image.

**Figure 4:**
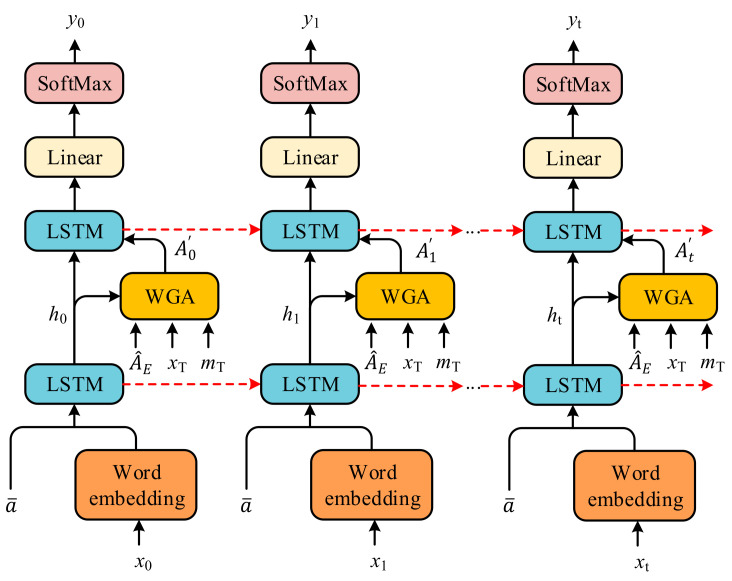
The framework of the decoder, which includes two LSTMs, a WGA module, and a prediction of word probability distribution.

In the decoder, we generated a sequence of captions ***y*** with the encoding results ${\hat{A}}_{E}$. The two-LSTM framework is composed of a language LSTM $LSTM^{lang}$ and an attention LSTM $LSTM^{Att}$, as shown in Figure 4.

We input the mean pooling visual vector $\bar{a}=\frac{1}{n}\sum {\hat{A}}_{E}$ and the *t*-th time step embedded word $W_{e}x_{t}$ to the attention LSTM $LSTM^{Att}$, which can be defined as

(10) $$h_{t}^{Att},\;m_{t}^{Att}=\;LSTM^{Att}\left(\left[W_{e}x_{t},\;\bar{a}\right],\;h_{t-1}^{Att},\;m_{t-1}^{Att}\right),$$

where t∈T, and $h_{T}^{Att},\;m_{T}^{Att}\in {\mathbb{R}}^{D}$ are the hidden state and memory cell of the $LSTM^{Att}$ respectively.

To make the WGA produce a marked effect in decoder, we inserted it between the $LSTM^{lang}$ and $LSTM^{Att}$ to guide the language model. As shown in Figure 4, $A_{t}^{\prime }$ can be obtained from $f_{\mathrm{WGA}}\left(.\right)$ fed with ${\hat{A}}_{E},\;W_{e}x_{T},\;m_{T}$ and $h_{t}^{Att}$, which is formulated as

(11) $${A^{\prime }}_{t}=f_{WGA}\left(h_{t}^{Att},\;{\hat{A}}_{E}^{K},\;{\hat{A}}_{E}^{V},\;W_{e}x_{T},\;m_{T}\;\right),$$

where *Q*, *K,* and *V* are replaced with $h_{t}^{Att}$ and double ${\hat{A}}_{E}$. In addition, $W_{e}x_{T},\;m_{T}$ are discussed after the language LSTM model.

The input to the $LSTM^{lang}$ is the concatenation of the WGA weighted feature vectors $A_{t}^{\prime }$ and the current hidden state of the $LSTM^{Att}$. Therefore, the $LSTM^{lang}$ can be presented as

(12) $$h_{t}^{lang},\;m_{t}^{lang}=\;LSTM^{lang}\left(\left[{A^{\prime }}_{t},\;h_{t}^{Att}\right],\;h_{t-1}^{lang},\;m_{t-1}^{lang}\right),$$

where t∈T and $h_{T}^{lang},\;m_{T}^{lang}\in {\mathbb{R}}^{D}$ are the hidden state and memory cell of the $LSTM^{lang}$, respectively.

Therefore, we can achieve a probability distribution $y_{t}$ of the caption prediction at time step *t*:

(13) $$p_{\theta }\left(y_{t}|y_{1:t-1}\right)=\mathrm{softmax}\left(W_{h}h_{t}^{lang}\right),$$

where $W_{h}\in {\mathbb{R}}^{D\times D}$, $y_{1:T}$ refers to the generated captions, and SoftMax is the activation function.

**Figure 5:**
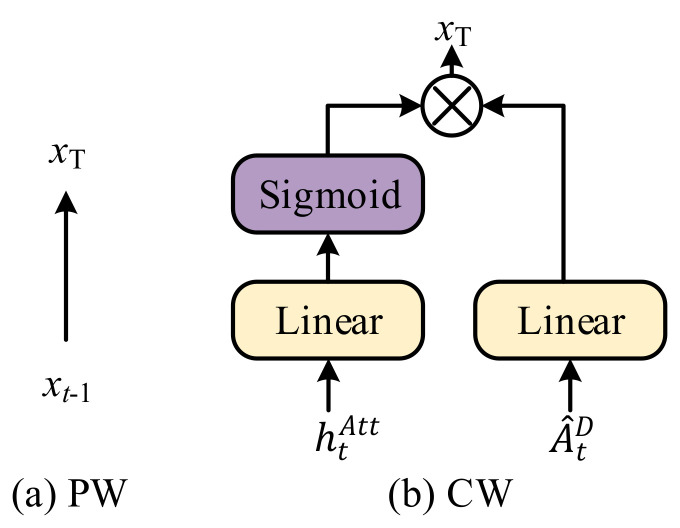
The illustration of PW and CW. (**a**) In PW, word information comes from the previous step and is applied briefly. (**b**) In CW, word information is provided from the current step attention LSTM and refined by linear transformation and sigmoid.

As stated earlier, we fed different $W_{e}x_{T},\;m_{T}$ into the WGA to realize different generation improvements, as shown in Figure 5.

**Previous word guided attention**. To better describe the entire scene, we made use of previous word information $W_{e}x_{t-1}$ and memory cell $m_{t-1}^{lang}$ (Figure 5a):

(14) $$W_{e}x_{t-1},\;m_{t-1}^{lang}\to W_{e}x_{T},\;m_{T}.$$

We believe that the word information from the previous step can protect the logical correctness for generation of the current word. Furthermore, the information is a summary of the previous attention region. It can guide the model to select the correct attention region for the current step, so that some neglected attention regions can be effectively utilized, and the WGA can cover more objects and relations.

**Current word guided attention**. For a more detailed description of the current attention region, we applied the current memory cell $m_{t}^{Att}$ of the $LSTM^{Att}$ and simulated generating the current word via $h_{t}^{Att}$ and gated linear units (Figure 5b):

(15) $${\hat{A}}_{t}^{D}=f_{Att}\left(h_{t}^{Att},\;{\hat{A}}_{E}^{K},\;{\hat{A}}_{E}^{V}\right),$$

(16) $$X_{c}=\left(W_{Att}{\hat{A}}_{t}^{D}+b^{Att}\right)\times \delta \left(W_{gate}h_{t}^{Att}+b^{gate}\right),$$

(17) $$X_{c},\;m_{t}^{Att}\to W_{e}x_{T},\;m_{T},$$

where ${\hat{A}}_{t}^{D}$ means the result of attended ${\hat{A}}_{E}$ by basic attention unit $f_{\mathrm{Att}}\left(.\right)$, $W_{Att}{,\;W}_{gate}\in {\mathbb{R}}^{D\times D},\;b^{*}\in {\mathbb{R}}^{D}$, and δ is a sigmoid function. We think that the current word information can help the model focus on the current attention region that is significant by weighting the feature vectors. Thus, the salience region can provide more details that are not only about objects but also deeper relations or status.

### 3.3. Training and Objectives

**Training with cross-entropy loss.** We first trained the image captioning model using cross-entropy loss *L_XE_*:

(18) $$L_{XE}\left(\theta \right)=-\sum_{t=1}^{T}\log\left(p_{\theta }\left(y_{t}^{*}|y_{1:t-1}^{*}\right)\right),$$

where $y_{1:T}^{*}$ refers to the ground truth of captions.

**Optimize using the CIDEr-D score.** Then, we followed the approach of self-critical sequence training (SCST) [30] to optimize the model:

(19) $$L_{RL}\left(\theta \right)=-E_{y_{1:\;T}\sim p_{\theta }}\left[r\left(y_{1:\;T}\right)\right],$$

where the reward *r*(.) is calculated by the metrics of Consensus-Based Image Description Evaluation score (CIDEr-D) [31]. The gradient is defined as

(20) $$\nabla_{\theta }L_{RL}\left(\theta \right)\approx -\left(r\left(y_{1:\;T}^{s}\right)-r\left({\hat{y}}_{1:\;T}\right)\right)\nabla_{\theta }\log p_{\theta }\left(y_{1:\;T}^{s}\right),$$

where $y^{s}$ is the result from the sampled probability, and $\hat{y}$ is the result of the greedy algorithm.

## 4. Experiments

### 4.1. Dataset

The proposed method was implemented on the MSCOCO [32] dataset. The MSCOCO dataset contains 123,287 images, including 82,783 training images and 40,775 validation images, with 5 captions for each. The Karpathy split [33] was adopted to obtain 113,287 images for training, 5000 images for validation, and 5000 images for testing. We collected words occurring more than 4 times in all sentences from the MSCOCO dataset and obtained a dictionary of 10,369 words. The metric we used to evaluate our method was Bilingual Evaluation Understudy score (BLEU-*N*) [34], which can be calculated as

(21) $$\mathrm{BLEU}-N=\min\left(1,\;e^{1-\frac{r}{c}}\right)\cdot e^{\frac{1}{N}\sum_{n=1}^{N}\log p_{n}},$$

where r and c denote ground truth captions and generated captions, respectively, and $p_{n}$ is the *n*-*gram* precision. In addition, we also adopted CIDEr-D [31], Metric for Evaluation of Translation with Explicit Ordering (METEOR) [35], Semantic Propositional Image Caption Evaluation (SPICE) [36], and Recall-Oriented Understudy for Gisting Evaluation (ROUGE-L) [37] to evaluate our method. These values calculate the similarity between the generated caption and the ground truth caption, and higher values represent better results. We computed this metrics with the public code (https://github.com/tylin/coco-caption, accessed on 25 September 2021) from the MSCOCO dataset.

### 4.2. Implementation Details

The Faster R-CNN model [2] trained on ImageNet [38] and Visual Genome [39] was exploited to extract bottom-up feature vectors from images. These vectors have 2048 dimensions and were transformed to 1024-D vectors to match the hidden size of the LSTM in the decoder. In the training phase with cross-entropy loss, we adopted an initial learning rate of 4e–4 decaying 0.8 every 2 epochs, and the ADAM optimizer was employed over a total of 30 epochs. For training with CIDEr-D score optimization, we set the initial learning rate to 4e–5 and decayed it by 50% when the performance of the validation split was never better in another 20 epochs. In addition, we set the image batch size to 10 during training, and the beam size was 2 while testing.

### 4.3. Quantitative Analysis

To validate the performance of our method, we gathered some results based on the Karpathy split test from other methods. These methods are based on certain well-known frameworks or improved attention, including LSTM [20], which encodes an image into CNN features and decodes them to a series of words using LSTM; SCST [30], which proposes a sequence train with evaluation metrics using reinforcement learning; Adaptive-Attention [27], which proposes an adaptive attention model with a visual sentinel; RFNet [40], which proposes a novel recurrent fusion network to exploit multiple information from encoders; UpDown [22], which applies two LSTMs to weight bottom-up image feature; research [41,42] that contributes new attention using semantic-enhanced image features and spatiotemporal memory, respectively; research [43] that provides special decoding phase improved by a ruminant mechanism; research [44] that leverages object attributes to structure linguistically-aware attention for the lack of high-level understanding; VRAtt-Soft [45], which proposes novel visual relationship attention via contextualized embedding for individual regions; and research [46] that extends the caption model by incorporating extra explicit knowledge from a memory bank. The results shown as percentage are presented in Table 1 and Table 2.

We report the performance of the methods with cross-entropy loss in Table 1, and it can be seen that our PW is superior to other methods in all metrics. CW can also achieve the same performance compared with others and is slightly better than PW. In addition, we present the comparison among methods trained with cross-entropy loss and optimized via CIDEr-D score optimization in Table 2. The results demonstrate that CW achieves the optimal performance among all methods, and PW is second best. Furthermore, we collect four models with different initial training parameters to perform ensemble evaluation and the comparison is in Table 3. Our method obtains satisfactory results in contrast to others.

### 4.4. Qualitative Analysis

We reported some examples of images and corresponding captions gathered from our PW, CW, the baseline, and ground truth. Note that we reimplemented the UpDown [22] model sharing the parameters of our models as a baseline. From Table 4, where we mark the improvements in blue, we found that the baseline rigidly describes the prominent objects without exact relations between objects and a detailed depiction of objects. Moreover, our models were superior in two ways: a) Our models focus on the whole image and obtain nearly all components in an image. For the first example, the baseline just recognizes “*women*” and a shallow relation but ignores others such as wine and glasses. In contrast, the captions from PW contain more objects and count more correctly, including “*wine*” and “*glasses*”, and CW also contains the background of “*room*”. The other three examples can confirm this conclusion. b) Our models can obtain the connections between objects in greater scope and depth with PW and CW. As seen in the first example, PW achieves objects of “*two women*” and “*wine glasses*” and then prefers to build the relationship between “*women*” and connect “*women*” with “*wine glasses*” by “*holding*”. Meanwhile, CW can guide the model in another direction. To use the same example, CW determines the relationship among “*women*”, “*wine*”, and “*glasses*” and then describes it with “*drinking*”. In other examples, we can find the same conclusion. PW and CW have these superiorities because they can guide the model to distribute attention for different purposes. As we can find in Table 4, PW and CW are experts in building relations between objects due to the self-attention and basic WGA. PW can determine how the model covers the objects in an image, and CW can more deeply assess the details of the current attention region, which we will show in Section 4.5.

### 4.5. Ablative Studies

To quantify the influence of our WGA models, we compared PW and CW against other methods with the same training phase. First, the UpDown method was defined as the baseline model, which adopted two LSTMs and attention to generate captions. Second, we employed the metrics of B@1, B@4, ROUGE-L, and CIDEr-D to evaluate the models trained after CIDEr-D score optimization. Finally, we refer to self-attention as **self-att**, the encoder phase as ***Enc***, and the decoder phase as ***Dec*** in Table 5.

**Effect of self-attention.** To evaluate the influence of self-attention, we successively extended the baseline with self-attention on the decoder and encoder. In the decoder, self-attention was located between the two-layer LSTM and became the backbone to build residual construction. From Table 5, we observe that replacing the original attention with self-attention brings benefits and improves the B@4 and CIDEr-D scores of the baseline by 0.9 and 2.0, respectively. In the encoder phase, we utilized self-attention to highlight the principal parts of the image. In Table 5, we can easily conclude that the weighted feature representations give the model effective impact. The results show that further self-attention improved the B@4 and CIDEr-D scores by 0.8 and 1.1, respectively.

**Effect of word guided attention.** We made further efforts to conduct experiments to test performance of the PW and CW modules. These two were designed following self-attention and constitute the WGA model during the decoding phase. In Table 5, we obtain a B@4 increase of 0.7 and a CIDEr-D increase of 1.8 for PW. On the other hand, CW ameliorated B@4 and CIDEr-D scores of **self-att(*Enc+Dec*)** by 0.7 and 1.4, respectively. Unfortunately, the B@4 and CIDEr-D scores were 38.8 and 126.2, respectively, when combining PW with CW by contacting themselves. We think that PW and CW guide the model in distinct directions and mistake the inference results. Even so, the basic word guided module still works, which is the reason why it is always better than **self-att(*Enc+Dec*)**.

To qualitatively present the influence of WGA, we visualized the sentences generated by the ablated models. Referring to Table 4, we present the ablation results in Table 6. As we can see, the sentence of an image was increasingly abundant from the baseline to PW/CW. The captions of PW/CW also confirmed their model characteristics. For example, PW and CW obtained different styles of improvement compared with the caption of **self-att(*Enc+Dec*)** in the last example. PW added a “*skis*” object and built the connection between “people” and “skis”, described as “*riding skis down*”. CW replaced “*A group of people*” with “*Two men*” for more detailed information and deep captioning.

## 5. Conclusions

In this paper, we propose a novel attention guided by word information (WGA) for image captioning, which is aimed at extracting more valuable information from the images. The proposed attention contains a novel word guiding weighting (WGW), which is built upon the extended word information, and a residual structure. Therefore, the WGA can provide the various semantic information to address the lack of objects and image details for the captioning model. After that, we propose different applications of WGA for the decoder and obtain the previous word guided attention (PW) and current word attention (CW) with different timing. We demonstrate that PW can expand the insight of the model to cover more objects in the image, and CW can focus on the current region to extract further information. More remarkably, we achieve competitive performance against other methods, and experimental results conclude that our proposed method has stability and universality.

In the future, we will explore how to fuse word information in the encoder to guide the captioning model. For the PW, the key is to find out the breakpoint in which the word information can be embedded. On the other hand, how to simulate word information in the current time step to construct CW is an issue. In addition, the gap between the image feature and word information needs to be solved.

## Figures and Tables

**Table 1:** The image captioning results of our method and others on the MSCOCO Karpathy test split with cross-entropy loss.

Method	BLEU-1	BLEU-2	BLEU-3	BLEU-4	METEOR	ROUGE-L	CIDEr-D	SPICE
LSTM [20]	-	-	-	29.6	25.2	52.6	94.0	-
SCST [30]	-	-	-	30.0	25.9	53.4	99.4	-
Adaptive-Attention [27]	73.4	56.6	41.8	30.4	25.7	-	102.9	-
RFNet [40]	76.4	60.4	46.6	35.8	27.4	56.5	112.5	20.5
UpDown [22]	77.2	-	-	36.2	27.0	56.4	113.5	20.3
Att2in+RD [43]	-	-	-	34.3	26.4	55.2	106.1	19.7
UpDown+STAM [41]	77.4	61.5	47.6	36.5	27.4	56.8	114.4	20.5
Ours: PW	77.4	61.5	47.7	36.8	28.1	57.3	117.0	21.2
Ours: CW	77.2	61.5	47.8	36.9	28.0	57.2	117.4	21.1

**Table 2:** The image captioning results of our method and others on the MSCOCO Karpathy test split after CIDEr-D score optimization.

Method	BLEU-1	BLEU-2	BLEU-3	BLEU-4	METEOR	ROUGE-L	CIDEr-D	SPICE
LSTM [20]	-	-	-	31.9	25.5	54.3	106.3	-
SCST [30]	-	-	-	34.2	26.7	55.7	114.0	-
RFNet [40]	79.1	63.1	48.4	36.5	27.7	57.3	121.9	21.2
UpDown [22]	79.8	-	-	36.3	27.7	56.9	120.1	21.4
Cai et al. [42]	80.0	64.3	49.6	37.5	28.2	58.2	126.0	21.8
UpDown+RD [43]	80.0	-	-	37.8	28.2	57.9	125.3	-
UpDown+STAM [41]	80.2	64.4	49.7	37.7	28.2	58.1	125.9	21.7
UpDown+LAT [44]	80.4	-	-	37.7	28.4	58.3	127.1	22.0
VRAtt-Soft [45]	80.2	63.3	48.7	37.3	28.4	61.4	121.8	21.8
UpDown+MA [46]	80.2	-	-	37.5	28.4	58.2	125.4	22.0
Ours: PW	80.4	65.1	50.8	39.1	28.7	58.7	127.6	22.2
Ours: CW	80.6	65.2	50.9	39.1	28.7	58.8	127.2	22.1

**Table 3:** The performance of our ensemble model.

Method	BLEU-4	METEOR	ROUGE-L	CIDEr-D	SPICE
SCST [30]	35.4	27.1	56.6	117.5	-
RFNet [40]	37.9	28.3	58.3	125.7	21.7
Yan et al. [47]	38.4	27.8	57.9	121.6	21.5
Ours: PW	39.6	28.7	59.1	128.3	22.2
Ours: CW	39.8	28.8	59.4	128.3	22.2

**Table 4:** Samples of image captions generated by our PW, CW, and baseline as well as ground truths.

Image	Captions
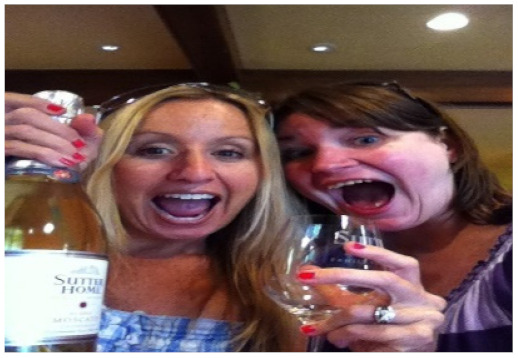	**Baseline:** A couple of women standing next to each other. **Our PW:** Two women standing next to each other holding wine glasses. **Our CW:** Two women drinking wine in a room. GT1: Two young women are sharing a bottle of wine. GT2: Two female friends posing with a bottle of wine. GT3: Two women posing for a photo with drinks in hand.
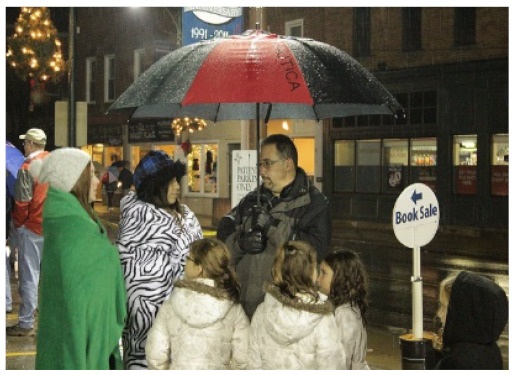	**Baseline:** A group of people walking down a street. **Our PW:** A group of people standing in the street with an umbrella. **Our CW:** A group of people standing under an umbrella. GT1: Several people standing on a sidewalk under an umbrella. GT2: Some people standing on a dark street with an umbrella. GT3: Some people standing on a dark street with an umbrella.
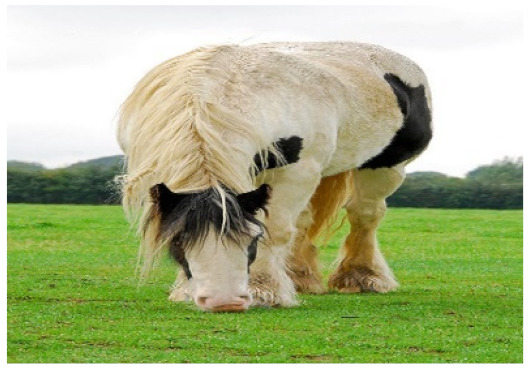	**Baseline:** A close up of a horse in a field. **Our PW:** A white horse standing in the grass in a field. **Our CW:** A white horse grazing in a field of grass. GT1: A horse eating grass in a green field. GT2: A while horse bending down eating grass. GT3: A tall black and white horse standing on a lush green field.
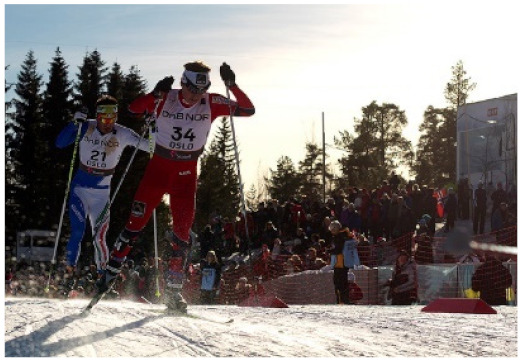	**Baseline:** A group of people on skis in the snow. **Our PW:** A group of people riding skis down a snow covered slope. **Our CW:** Two men are skiing down a snow covered slope. GT1: Two cross country skiers heading onto the trail. GT2: Two guys cross country ski in a race. GT3: Skiers on their skis ride on the slope while others watch.

**Table 5:** Ablation studies for our method. All results are obtained after CIDEr-D score optimization.

Model	BLEU-1	BLEU-4	ROUGE-L	CIDEr-D
Baseline	79.4	36.7	57.6	122.7
**+self-att(*Dec*)**	80.0	37.6	58.0	124.7
**+self-att(*Enc*+*Dec*)**	79.9	38.4	58.4	125.8
Full: PW	80.4	39.1	58.7	127.6
Full: CW	80.6	39.1	58.8	127.2

**Table 6:** Visualization of the generated captions of the ablated models, where the colored words are the improvements from the previous caption.

Image	Captions
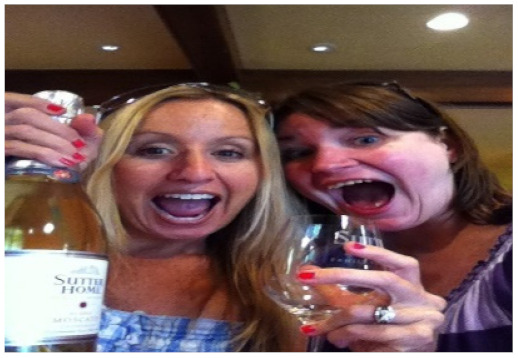	**Baseline:** A couple of women standing next to each other. **+self-att(Dec):** A couple of women standing next to each other. **+self-att(Enc+Dec):** Two women are holding wine glasses in a room. **Our PW:** Two women standing next to each other holding wine glasses. **Our CW:** Two women drinking wine in a room.
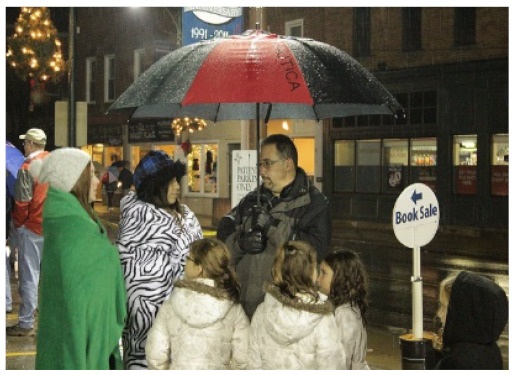	**Baseline:** A group of people walking down a street **+self-att(Dec):** A group of people standing in the street. **+self-att(Enc+Dec):** A group of people standing with an umbrella. **Our PW:** A group of people standing in the street with an umbrella. **Our CW:** A group of people standing under an umbrella.
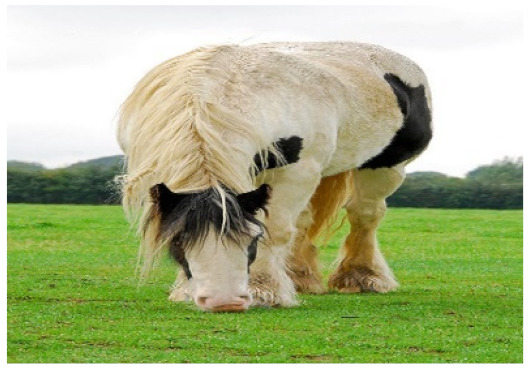	**Baseline:** A close up of a horse in a field. **+self-att(Dec):** A horse standing in a field. **+self-att(Enc+Dec):** A horse in the grass in a field. **Our PW:** A white horse standing in the grass in a field. **Our CW:** A white horse grazing in a field of grass.
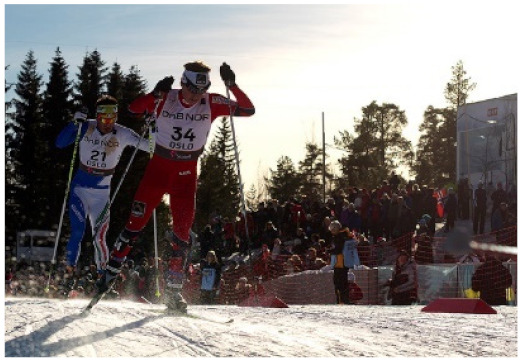	**Baseline:** A group of people on skis in the snow. **+self-att(Dec):** A man riding skis in the snow. **+self-att(Enc+Dec):** A group of people skiing down a snow covered slope. **Our PW:** A group of people riding skis down a snow covered slope. **Our CW:** Two men are skiing down a snow covered slope.

## Data Availability

Publicly available datasets were analyzed in this study. This data can be found here: https://cocodataset.org/ (accessed on 25 September 2021).
